# Recovery of preoperative absolute knee extension and flexion strength after ACL reconstruction

**DOI:** 10.1186/s13102-020-00222-8

**Published:** 2020-12-10

**Authors:** Ramana Piussi, Daniel Broman, Erik Musslinder, Susanne Beischer, Roland Thomeé, Eric Hamrin Senorski

**Affiliations:** 1 Sportrehab Sports Medicine Clinic, Stampgatan 14, SE-411 01 Gothenburg, Sweden; 2 Sahlgrenska Sports Medicine Center, Gothenburg, Sweden; 3 Unit of Physiotherapy, Department of Health and Rehabilitation, Institute of Neuroscience and Physiology, Sahlgrenska Academy, University of Gothenburg, Box 455, SE-405 30 Gothenburg, Sweden

**Keywords:** Knee, Anterior cruciate ligament, Evaluation, Limb symmetry index

## Abstract

**Background:**

The recovery of muscle function after an Anterior Cruciate Ligament (ACL) reconstruction is most commonly reported as limb-to-limb differences using the Limb Symmetry Index (LSI), which is not free from limitations. The purpose of this study was to compare the proportion of patients who recover their Preoperative Absolute Muscle Strength (PAMS) 8 and 12 months after ACL reconstruction with the proportion of patients who recover their symmetrical knee strength. A secondary aim was to assess the relationship between psychological Patient-Reported Outcomes (PROs) and recovering PAMS at 8 and 12 months after ACL reconstruction and rehabilitation.

**Method:**

Preoperative, 8- and 12-month results from quadriceps and hamstring strength tests and PROs for 117 patients were extracted from a rehabilitation registry. Individual preoperative peak torques from strength tests were compared with results from the 8- and 12-month follow-ups respectively. Patients were defined as having recovered their PAMS upon reaching 90% of their preoperative peak torque for both quadriceps and hamstring strength. Patients were defined as having recovered their LSI upon reaching a value ≥90% when comparing the results for their injured knee with those of their healthy knee. Correlations between the recovery of PAMS and PROs at 8 and 12 months were analyzed.

**Results:**

There was no difference in the proportion of patients who recovered their PAMS compared with patients who recovered their LSI. In all, 30% and 32% of the patients who recovered their LSI had not recovered their PAMS at 8 months and 12 months respectively. In the patients who had recovered their PAMS, 24% and 31% had not recovered their symmetrical LSI at 8 months and 12 months respectively. There was no significant correlation between the recovery of PAMS and psychological PROs.

**Conclusion:**

The use of both PAMS and LSI provides more detailed information on the recovery of muscle strength after ACL reconstruction. The recovery of PAMS was not correlated with psychological traits, which implies that both PROs and PAMS are important when evaluating patients after ACL reconstruction.

**Trial registration:**

This trial was not registered.

## Background

An Anterior Cruciate Ligament (ACL) injury is a common sports-related knee injury [11]. After a rupture of the ACL, a period of rehabilitation consisting of neuromuscular and strength training is warranted [27]. Post-injury rehabilitation is important since not all patients benefit from early surgery, sometimes referred to non-copers [19]. Furthermore, post-injury rehabilitation might lead to patients resuming pre-injury activity without the need for surgery, and might restore some knee stability in patients, showing better outcomes after ACL reconstruction [27]. After an ACL reconstruction and individualised rehabilitation, an important goal for most patients and healthcare providers is a safe return to sport (RTS) [33]. Approximately 60% of patients RTS within 2 years of ACL reconstruction, of which 30% go on to suffer a second ACL injury [3], or meniscal or cartilage injuries [15]. Passing batteries of strength and hop tests before RTS can reduce this risk of re-injury after ACL reconstruction [4, 15]. Furthermore, specific cut-offs for poor Patient-Reported Outcomes (PROs), reflecting a lower psychological readiness to return to sport, appear to correlate with a second ACL injury [20]. For this reason, evaluating patients’ muscle function and psychological well-being after ACL reconstruction could be beneficial to clinical practice.

Burgi et al. [8] reported that the time from reconstruction is the most frequently used criterion in the literature for the RTS decision-making process after ACL reconstruction. However, using the results of muscle function tests and psychological PROs as the criterion for determining a safe RTS is becoming more common [2, 4, 12, 22, 25, 36]. The results of muscle function tests after ACL injury, regardless of treatment, are often reported as limb-to-limb differences, i.e. the proportional recovery of the injured limb compared with the uninjured limb, using the Limb Symmetry Index (LSI) [1, 2]. This is, however, not free from limitations, which include the fact that patients commonly suffer from loss of strength in the uninjured limb, as well as the injured limb, entailing the risk of overestimating recovery [30]. In these cases, using the LSI suggests symmetrical muscle function based on bilateral weakness, thereby increasing the risk of sending an athlete back to sport too early [38]. Accordingly, whether the use of the LSI as an RTS criterion is stringent enough has been questioned [7, 38]. Adding absolute strength measurements as outcomes after ACL reconstruction could possibly provide more detailed information on the recovery of strength [31].

The recovery of symmetrical strength (LSI) has been linked with higher knee-related self-efficacy [23, 29] and a higher readiness to RTS [9, 37] measured with PROs, and although novel attempts have been made to determine the effectiveness of preoperative absolute muscle strength (PAMS) as an RTS criterion, the relationship between the recovery of PAMS and psychological PROs remains unclear. Gokeler et al. [14] found that patients who underwent ACL reconstruction did not perform as well as healthy controls in hop tests 7 months after surgery, although they achieved symmetrical LSI in hop tests. In addition, Wellsandt et al. [38] showed that, after ACL reconstruction, patients struggled to achieve preoperative results in a battery of tests. Therefore, a better understanding of the relationship between PAMS and psychological PROs is warranted and could further help clinicians determine the recovery of patients after ACL reconstruction.

The purpose of this study was to compare the proportion of patients who recover their preoperative absolute muscle strength (PAMS) 8 and 12 months after ACL reconstruction with the proportion of patients who recover their symmetrical knee strength. In addition, the study aimed to assess the relationship between psychological PROs and recovering PAMS at 8 and 12 months after ACL reconstruction and rehabilitation.

## Method

The present study used data from Project ACL, extracted on 13 July 2019. Project ACL is a rehabilitation-specific registry located in Sweden for patients with an ACL injury. The data consist of the results of muscle function tests and PROs, which are collected prospectively, starting with ACL injury or reconstruction as a baseline and thereafter at predefined follow-ups: 10 weeks, 4, 8, 12, 18 and 24 months, 5 years and every fifth year thereafter. The registry has previously been described in detail [6, 16] and ethical approval has been obtained from the Regional Ethical Review Board in Gothenburg, Sweden (registration numbers: 265–13, T023–17).

### Patients

All patients with a unilateral ACL injury treated with primary ACL reconstruction and rehabilitation were considered eligible for inclusion to this study. Patients were excluded after having recorded more than one ACL injury, not having performed strength tests preoperatively and not participating at both the 8- and the 12-month follow-ups after ACL reconstruction.

### Strength tests

**Fig. 1:**
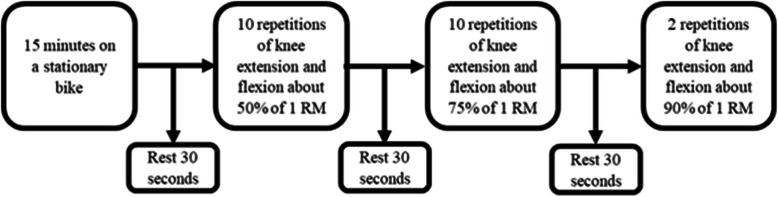
Standardized warm-up procedure

Quadriceps and hamstring peak torque were measured concentrically with an isokinetic dynamometer (Biodex System 4; Biodex Medical System, Shirley, New York, USA) [32] at an angular speed of 90°/second. Quadriceps and hamstring strength testing with the Biodex is reliable when it comes to measuring isokinetic muscle strength, Intraclass Correlation Coefficient = 0.95 [10]. Quadriceps and hamstring strength (peak torque) was assessed unilaterally in a seated position, (approximatively 110° of hip flexion) with shoulders, waist, thigh and distal lower leg being strapped and arms crossed over the chest. The centre of the knee joint was aligned to the centre of movement axis of the dynamometer at 90° of knee flexion, while at rest. Knee extension (quadriceps) peak torque was measured from 90° to 0° of flexion, while knee flexion (hamstring) peak torque was measured from 0° to 90° of flexion. After a standardized warm-up (Fig. 1), 3 maximum repetitions of knee extension, instantly followed by knee flexion, were performed with 30 s of rest between each repetition. Patients were offered the opportunity of doing an additional fourth repetition if they felt they could do one more. The peak torque in Newton / meters was used for analysis.

### Patient-reported outcomes

The PROs used in this study were the Knee Self-Efficacy Scale (K-SES), future subscale (K-SES_future_) and the ACL Return to Sport after Injury scale (ACL-RSI).

The K-SES was developed to measure knee-related self-efficacy in patients with an ACL injury [28]. The original scale has good reliability (ICC = 0.75) and good validity [28]. The subscale future of the K-SES consists of 4 questions where patients rate from 0 (not at all certain) to 10 (very certain) how certain they feel about their knee in the future. The results from each question are added together and divided by four to obtain a mean score for each patient. The maximum score is 10 and reflects the highest future perceived knee-related self-efficacy. The preoperative scores for the K-SES_future_ were used for analysis in this study. The future subscale of the K-SES was chosen, as it has been shown to predict the recovery of LSI 12 months after an ACL reconstruction [29].

The ACL-RSI was chosen since it is considered to have high methodological quality for patients with an ACL injury [12]. The ACL-RSI has good homogeneity (Cronbach’s alpha = 0.95) and good validity to assess psychological readiness to RTS [35]. In this study, the 12-item version was used. The scale is graded from 0 to 10, where 0 reflects the lowest readiness to RTS and 10 the highest [34, 35]. The responses from each item are added together to obtain a total score (range 0–120). Data from the ACL-RSI were analyzed for the 8- and 12-month follow-ups.

### Outcomes

The recovery of PAMS was the primary outcome of this study. The greatest preoperative peak torque for quadriceps and hamstring strength recorded from any limb from each individual patient was used as a reference for individual recovery. Preoperative peak torque for quadriceps and hamstrings for each individual patient were compared with the patient’s quadriceps and hamstring peak torque at the 8- and 12-month follow-up respectively. Patients were defined as having recovered their PAMS upon reaching 90% of their preoperative peak torque for both quadriceps and hamstring strength.

The proportion of patients that had recovered their PAMS was compared with the proportion of patients that had recovered their symmetrical muscle strength (defined as LSI ≥ 90%, which is considered consensus for recovered symmetrical muscle strenght after ACL injury and reconstruction [18]). Upon failing to recover their PAMS, a further analysis was carried out in order to determine which strength test the individual patient had not passed.

The correlations between the recovery of PAMS and ACL-RSI scores at 8 and 12 months and the preoperative scores on the K-SES_future_ were analyzed as secondary outcomes.

### Statistics

Statistical analyses were performed with the Statistical Product and Service Solutions (IBM Corp. Released 2017. IBM SPSS Statistics for Windows, Version 25.0. Armonk, NY: IBM Corp.). The Chi-Square test was used to compare the proportion of patients that had recovered their PAMS and LSI respectively. The Chi-Square test was used to compare the proportion of patients who did not recover the different parameter of PAMS, that is, quadriceps or hamstring strength. For all proportions, 95% confidence intervals (CI) were provided. The point biserial correlation was used in the correlation analyses of recovery of PAM and PROs. Correlation coefficients were defined as weak (r = 0.00–0.39), moderate (r = 0.40–0.69), strong (r = 0.70–0.89) and very strong (r = 0.90–1.00) [24]. A significance level of 95% was used.

## Results

**Fig. 2:**
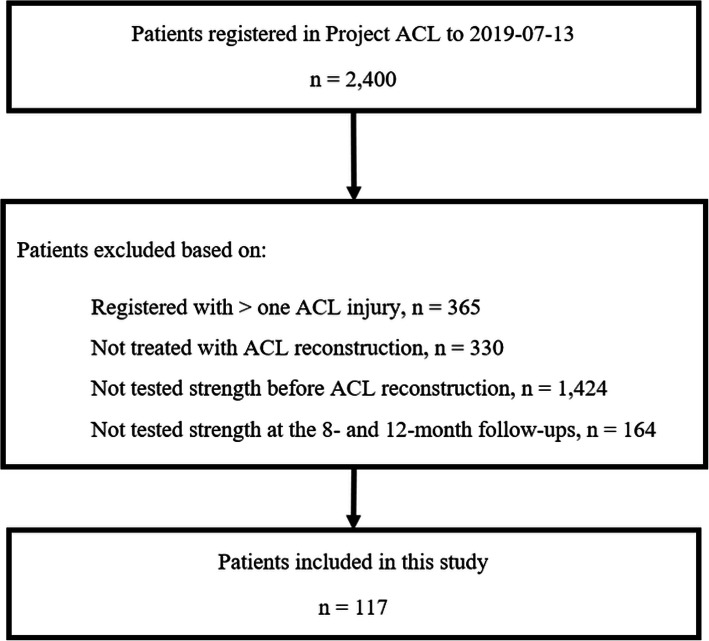
Flowchart of included and excluded patients

A total of 117 patients (men 33%) were included in the study (Fig. 2).

The patients were 30 ± 12 years old on average at the time of ACL reconstruction. Most patients (91%) received a hamstring tendon autograft (Table 1).

**Table 1:** Patient demographics, means and standard deviations

	Total	Men	Women
Patients, n (%)	117 (100)	39 (33)	78 (77)
Age, years	29.8 ± 11.7	32.3 ± 12.3	28.5 ± 11.3
Weight, kg	71 ± 13.7	85 ± 17.1	65 ± 11.1
Height, cm	173 ± 9.9	183 ± 6.1	168 ± 6.9
BMI, kg/height cm^2^	24 ± 3.2	26 ± 6.2	23 ± 3.1
Tegner, median (min-max)	8 (2–10)	8 (3–10)	8 (2–10)
Graft choice			
Hamstring, n (%)	90.6%	89.7%	91.0%
Patellar, n (%)	6.8%	10.3%	5.1%
Other, n (%)	2.6%	–	3.9%

*cm* centimeters; *kg* kilograms; *n* number; *Tegner* Tegner Activity Scale

**Fig. 3:**
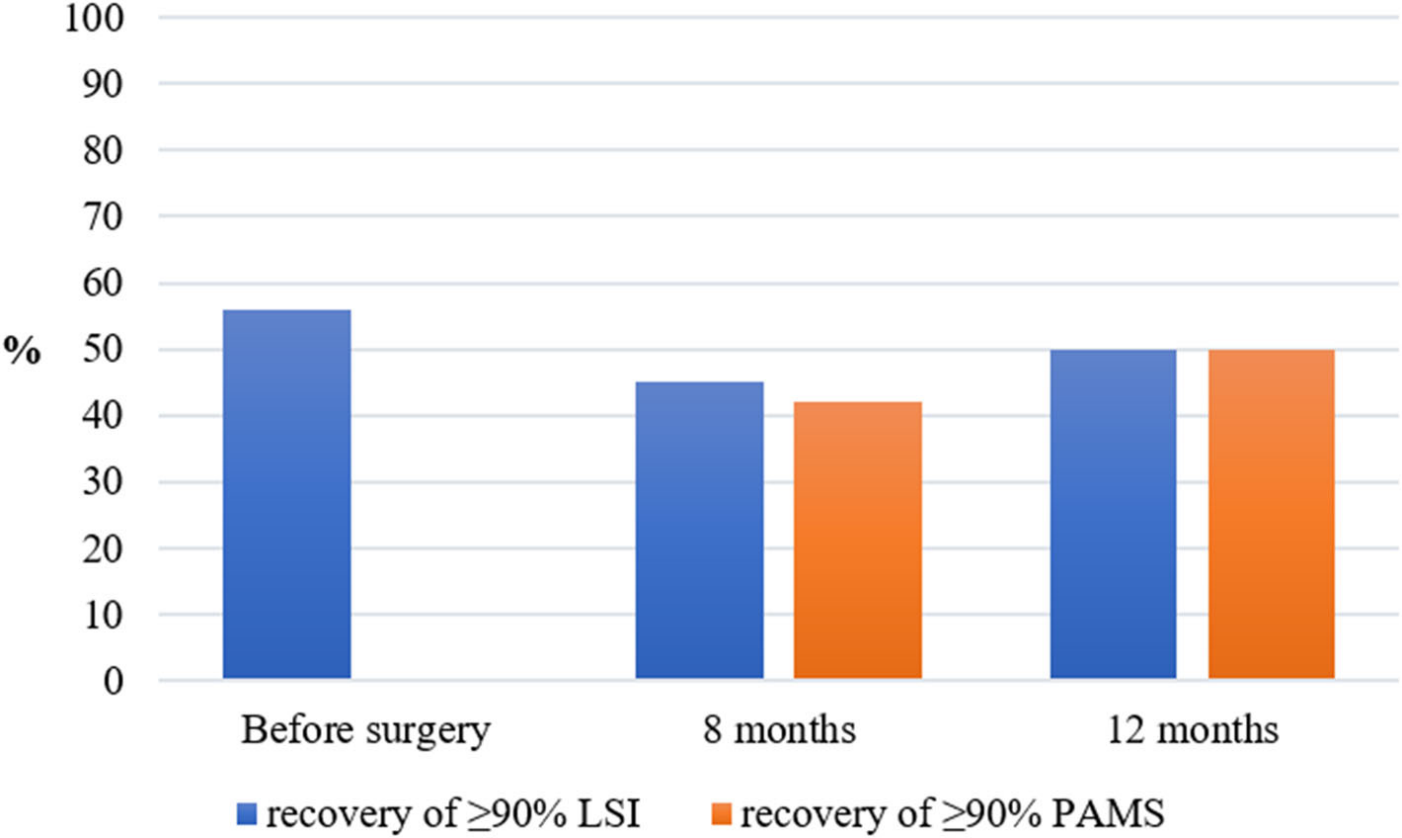
Proportion of patients recovering their Limb Symmetry Index and Preoperative Absolute Muscle Strength

In all, 56% (CI 47–66%) of patients had pre-surgery LSI values ≥90%. Further, 45% (CI 36–54%) of patients recovered LSI, and 42% (CI 41–60%) recovered PAMS at 8 months. At the 12 months follow up, 50% (CI 33–51%) of patients recovered LSI and 50% (40–59%) recovered PAMS. There was no difference in the proportion of patients who recovered their LSI or PAMS, at either 8 or 12 months after ACL reconstruction (Fig. 3).

In all, 30% (CI 18–44%) and 32% (CI 20–45%) of the patients who recovered their LSI had not recovered their PAMS at 8 months and 12 months, respectively. In the patients who had recovered their PAMS, 24% (CI 13–38%) and 31% (CI 19–43%) had not recovered their symmetrical LSI at 8 months and 12 months respectively.

There was no significant correlation between either the recovery of PAMS and ACL-RSI (8 months, r = 0.001; 12 months, r = 0.066) or PAMS and K-SES future (8 months, r = 0.086; 12 months, r = 0.049).

In patients who had recovered their LSI but had not recovered their PAMS, the most common reason was not having recovered their absolute strength for hamstrings at the 12-month follow-up (27% of patients had not recovered, *p* = 0.001) (Table 2).

**Table 2:** Numbers and proportions of patients recovering their LSI but not recovering their PAMS

n (%)	Recovered LSI	Not recovered PAMS quadriceps	Not recovered PAMS hamstrings	*p* value †
8 months	56 (45%) (CI 36–54%)	6 (11%) (CI 5–19%)	12 (22%) (CI 7–23%)	0.056
12 months	59 (50%) (CI 33–51%)	7 (11%) (CI 2–12%)	16 (27%) (CI 9–28%)	0.001

*n* number; *PAMS* Preoperative Absolute Muscle Strength; †, Chi Square test, *CI* Confidence Interval

## Discussion

The main finding in this study was that there was no difference in the proportion of patients who had recovered their PAMS compared with patients who recovered their LSI 8 and 12 months after ACL reconstruction. Furthermore, there was no significant correlation between the recovery of PAMS and psychological readiness for RTS or future knee-related self-efficacy. As a result, psychological status, reported with PROs, and the recovery of PAMS appear to cover two different dimensions of patient recovery. In addition, our results indicated that patients who recover their LSI, but not their PAMS, most frequently fail to recover their preoperative hamstring strength 12 months after ACL reconstruction (27% versus 11% for quadriceps, *p* = 0.001).

Despite the similar proportion of patients recovering their PAMS and LSI (45% and 42% at 8 months, 50% in both groups at 12 months), there were differences in terms of which individual patients recover their PAMS and LSI. At the 12-month follow-up, about one third of the patients who had recovered their LSI had not recovered their PAMS and, conversely, about one third of the patients who had recovered their PAMS had not recovered their LSI. These results infer that approximately one third of patients who recover their symmetrical muscle strength are weaker than before the ACL reconstruction and that about one third of patients who recover their absolute muscle strength do not have symmetrical muscle strength. The main reason for not recovering PAMS was not having recovered preoperative hamstring strength. It is known that harvesting the hamstring tendon as an autograft results in hamstring strength deficits, [5] which likely contributed to the present results. This is not the first study to compare preoperative absolute strength after ACL reconstruction. Wellsandt et al. [38] investigated 70 patients 6 months after ACL reconstruction and reported that only about 30% recovered their pre-surgery absolute strength. Differences in time to follow-ups and definitions of PAMS make comparisons of the present study with the study by Wellsandt et al. [38] difficult. However, Wellsandt et al. [38] reported that approximately one in three patients who recovered their symmetry (LSI) did not recover their preoperative muscle strength, which is in line with the results of the present study. Both lower levels of symmetry in the quadriceps and hop tests, [15] and of absolute muscle strength, [17] can increase the risk of primary and secondary ACL injury. The results of the present study suggest that PAMS and LSI provide two different aspects of recovery. For this reason, adding the assessment of PAMS can be a useful complement when determining the recovery of patients treated with ACL reconstruction. However, future research is needed to confirm the role played by PAMS in secondary ACL injury reduction.

Since the recovery of PAMS has not been studied to the same extent as LSI, there is no clear consensus on how to define PAMS and which threshold to use to define recovery. Defining a recovery of PAMS by using ≥ 90% as a cut-off may be too low. Normally, an ACL injury is followed by a period of inactivity, which might lead to the loss of thigh and calf muscle mass and strength [13]. It may therefore be easier for patients to recover preoperative strength that is lower than the muscle strength they actually possessed at the time of injury. Additionally, the muscle strength that the patient possessed at the time of injury might not have been sufficient, as stronger muscles in lower extremities provide protection from primary ACL injury [21]. In order to reduce the risk of subsequent ACL injuries, PAMS cut-off values higher than > 90% should be considered.

### Limitations

The availability of data to determine PAMS is one limitation of this study. Many patients are enrolled in Project ACL after being treated with an ACL reconstruction. In the over 2000 patients available for inclusion, 1424 (58%) had not tested their strength preoperatively. Another limitation of our study concerns pre-operative rehabilitation. We do not know whether patients enrolled in this study performed pre-operative rehabilitation or not, or the quality of their rehabilitation. A further limitation might be the preoperative loss of strength in the injured limb [13]. In the present study, we accounted for this limitation by taking the highest preoperative peak torque for quadriceps and hamstring strength recorded from any limb, for each patient, as a reference for recovery, regardless of whether it was taken from the injured limb or the uninjured limb. Another limitation relates to the method used for strength testing (seated isokinetic dynamometer). The isokinetic dynamometer measures knee extensors and knee flexors concentric peak torque at an angular velocity of 90 ^o^/sec from a seated position and might therefore not reflect the full complexity of knee extensors and knee flexors muscle cooperation when performing a standing sport task in motion.

In our cohort, 77% of the patients (*N* = 78) were women. This is somewhat higher compared with the general population of patients with ACL injuries in Sweden, where 46% are women and 54% are men [11]. It is not known why more women had tested their strength before ACL reconstruction in the cohort, which limits the generalizability of our results. However, a recent systematic review [26] reported no between-sex differences in the results of strength testing expressed as the LSI for hamstrings and quadriceps in patients after an ACL reconstruction. The results were based on 8 studies, where no study reported significant differences between the sexes in strength testing expressed as the LSI.

In addition, the patients included in this study, as well as in Project ACL, are a population with a generally high level of physical activity, with a median of 8 on the Tegner Activity Scale, which is equivalent to basketball, handball and triple jump. The results may therefore not be applicable to a population with lower physical activity levels or to patients who have not undergone ACL reconstruction. It should also be noted that 90% of the patients in the present study underwent ACL-reconstruction using hamstring-graft which can explain the difficulty of recovering hamstring strength compared to quadriceps strength [5].

## Conclusion

There was no difference in the proportion of patients who recovered their PAMS compared with their LSI, although, in approximately one third of the cases, patients who recovered symmetry had not recovered their preoperative absolute muscle strength and vice versa. The use of both preoperative absolute muscle strength and symmetrical muscle strength therefore provides more details relating to the recovery of muscle strength after ACL reconstruction. The recovery of PAMS was not correlated with psychological traits, which implies that both PROs and PAMS are important when evaluating patients after ACL reconstruction.

## Data Availability

The dataset used and/or analyzed during the current study are available from the corresponding author in response to a reasonable request.

## Abbreviations

ACL: Anterior Cruciate Ligament
ACL-RSI: ACL Return to Sport after Injury scale
K-SES: Knee Self-Efficacy Scale
LSI: Limb Symmetry Index
N: Number
PAMS: Preoperative Absolute Muscle Strength
PROs: Patient-reported Outcomes
RM: Repetition Maximum
RTS: Return to Sport
